# Associations between pre-pregnancy body mass index and gestational weight gain with pregnancy outcomes in women with polycystic ovary syndrome

**DOI:** 10.1186/s13098-020-00595-3

**Published:** 2020-10-08

**Authors:** Lirui Zhang, Wei Zheng, Cheng Liu, Xin Liang, Li Zhang, Zhihong Tian, Guanghui Li

**Affiliations:** Division of Endocrinology and Metabolism, Department of Obstetrics, Beijing Obstetrics and Gynecology Hospital, Capital Medical University, No 251, Yaojiayuan Road, Chaoyang District, Beijing, 100026 China

**Keywords:** Pregnancy, Polycystic ovary syndrome, Body mass index, Gestational weight gain, Pregnancy outcome

## Abstract

**Background:**

The influence of pre-pregnancy body mass index (BMI) and gestational weight gain (GWG) on perinatal outcomes of women with polycystic ovary syndrome (PCOS) remains unclear. Therefore, we explored how the above indicators influence pregnancy outcomes in women with PCOS.

**Methods:**

A retrospective study was conducted involving the baseline characteristics, laboratory data, and pregnancy outcomes of 722 pregnant women with PCOS. Subjects were grouped in a way to find out risks in their pregnancy outcomes. Multivariable logistic regression analysis was performed to investigate how BMI and GWG were associated with perinatal outcomes.

**Results:**

Among women with PCOS, underweight increased the risk of small for gestational age (SGA) (OR 12.35, 95% CI 3.56–42.82), but reduced the risk of large for gestational age (LGA). Overweight but not obese women were more susceptible to developing preeclampsia (PE) than women with normal weight. In PCOS women with BMI < 25 kg/m^2^ before pregnancy, inadequate GWG was a protective factor for gestational hypertension (GH) and postpartum hemorrhage (PPH), excessive GWG exhibited a positive correlation with LGA. But in PCOS women with BMI ≥ 25 kg/m^2^, excessive GWG increased the probability of undergoing a cesarean section. Inadequate GWG did not reduce the likelihood of LGA in women with BMI ≥ 25 kg/m^2^, and excessive GWG did not reduce the probability of SGA in women with BMI < 25 kg/m^2^.

**Conclusion:**

The impacts of pre-pregnancy BMI, GWG on maternal and infant outcomes among PCOS women are similar to reported results in general pregnant women. However, some unique trends were also observed in PCOS women. While the underweight factor significantly increased the risk of SGA birth, overweight but not obesity was correlated with the risk of PE. Inadequate GWG was a protective factor for GH and PPH only in women with pregestational BMI < 25 kg/m^2^. Inadequate GWG did not reduce the probability of LGA in women with BMI ≥ 25 kg/m^2^, and similarly, excessive GWG did not reduce the probability of SGA in women with BMI < 25 kg/m^2^. Overall, these findings indicate that women with PCOS should begin weight management before pregnancy.

## Background

Polycystic ovary syndrome (PCOS), a common heterogeneous female endocrinopathy affecting approximately 5 to 20% of women of childbearing age worldwide [1], is characterized by hyperandrogenemia, hyperinsulinemia, and insulin resistance [2]. In recent years, the advancement in assisted reproductive technology has significantly increased the chances of pregnancy in PCOS women. However, this condition has increased the risk of complications during pregnancy or delivery, such as the prevalence of gestational diabetes mellitus (GDM), preeclampsia (PE), and premature delivery [3]. For instance, women with PCOS are more susceptible to overweight/obesity and experience higher GWG, than their normal counterparts [4, 5]. This may be, in part, due to neurohormonal gut-brain interactions in women with PCOS [6]. GWG in women is crucial to optimize maternal, fetal, and neonatal health, with previous studies associating inappropriate pre-pregnancy BMI and GWG with higher risks of adverse neonatal and maternal outcomes in general pregnant women [7–10]. However, few studies have described the impact of both pre-pregnancy BMI and GWG on maternal and infant outcomes of these special individuals. In light of this, our study investigated the effect of pre-pregnancy BMI and GWG on pregnancy outcomes of PCOS women, with the view of generating vital information to guide proper weight management before or during pregnancy and reduce related adverse maternal and infant outcomes.

## Materials and methods

### Study subjects

The present study was a retrospective cohort study comprising 722 PCOS women who established a medical record for receiving healthcare in the first trimester of pregnancy and delivered live-born singletons at the Beijing Obstetrics and Gynecology Hospital, Capital Medical University between July, 2017 and July, 2019. Participants were included in the study if they; (i) were women diagnosed with PCOS; (ii) were pregnant; (iii) were aged between 18 and 45 years; and (iv) had a singleton pregnancy. On the other hand, subjects were excluded if they; (i) were females with multiple pregnancies; (ii) had pre-existing hypertension, diabetes, acute and chronic heart, liver, kidney disease, or other serious diseases; (iii) exhibited fetal chromosomal abnormalities or major birth defects and; (iv) did not have complete clinical data. The study received ethical approval from the ethics committee of the Beijing Obstetrics and Gynecology Hospital affiliated with Capital Medical University, with all the participants signing informed consent documents prior to inclusion.

### Study design

PCOS diagnosis was based on the revised 2003 Rotterdam Criteria, which requires the presence of 2 of the following 3 criteria: (i) oligo- or anovulation, (ii) clinical and, or biochemical signs of hyperandrogenism, and (iii) polycystic ovaries and exclusion of other etiologies (congenital adrenal hyperplasia, androgen-secreting tumors, Cushing’s syndrome) [11]. We used the electronic medical record system of the hospital to collect the patient-level variables, such as standard demographic information (age, pre-pregnancy height and weight, GWG, number of prior pregnancies, parity, and family history) and relevant maternal and infant outcomes, as well as laboratory data. Pre-pregnancy BMI, calculated as weight divided by height squared (kg/m^2^) using self-reported pre-pregnancy weight, was used to categorize the subjects into 4 groups, according to World Health Organization (WHO)’s guidelines. Based on these criteria, individuals with a BMI of < 18.5, 18.5–24.9, 25.0–29.9, and ≥ 30 kg/m^2^, were classified as underweight, normal weight, overweight, and obese, respectively. Maternal GWG, calculated as weight before delivery minus the pre-pregnancy weight, was used to group the subjects into within or above the target as recommended by the Institute of Medicine (IOM). Based on these guidelines, different weight gain intervals exist based on pregestational BMI (Table 1). Weight gains of 12.5–18, 11.5–16, 7–11.5, and 5–9 kg are recommended for underweight, normal weight, overweight, and obese women, respectively. Gestational weight gains below or above the recommended threshold were defined as inadequate or excessive weight gains, respectively. Then, the association between maternal pre-pregnancy BMI, GWG clinical categories, and the risk of some perinatal outcomes among PCOS women, which have been rarely investigated, were explored in our study.

**Table 1:** Recommendations for total weight gain during pregnancy, by pregnancy body mass index, according to the guidelines of the Institute of Medicine and National Research Council (2009)

Pre-pregnancy weight category	Body mass index (kg/m^2^)	Recommended range of total weight (kg)
Underweight	< 18.5	12.5–18.0
Normal weight	18.5–24.9	11.5–16.0
Overweight	25.0–29.9	7.0–11.5
Obese	≥ 30	5.0–9.0

### Pregnancy outcome

Gestational hypertension (GH) was defined as de novo hypertension, if an individual exhibited a systolic blood pressure ≥ 140 mmHg and, or diastolic blood pressure ≥ 90 mmHg, after 20 weeks of pregnancy [12], whereas preeclampsia (PE) was defined by the onset of hypertension and proteinuria, after 20 weeks of gestation [13]. Gestational diabetes mellitus (GDM) diagnosis was based on the result of a standard 75 g oral glucose tolerance test between 24 and 28 weeks of gestation, if any one of the following criteria was met: plasma glucose ≥ 5.1 mmo/L, ≥ 10.0 mmol/L, and ≥ 8.5 mmol/L for fasting, 1 and 2 h respectively. Postpartum hemorrhage (PPH) was defined as blood loss ≥ 500 mL within 24 h of delivery. Small for gestational age (SGA) was defined as a birth weight below the 10th percentile for gestational age and gender, whereas large for gestational age (LGA) was defined as a birth weight above the 90th percentile for gestational age and gender [14]. Lastly, macrosomia was defined as a birth weight ≥ 4000 g, whereas assisted vaginal delivery was defined as forceps or vacuum and assisted breech delivery.

### Statistical analyses

All data were evaluated using the SPSS 23.0 software, and the results were presented as means ± standard deviations (SD) of the means. Enumeration data were analyzed using the Chi-square test, multivariable logistic regression analysis was applied to assess the correlation between BMI, and GWG with pregnancy outcomes, after controlling for relevant confounding factors, such as maternal age, height, pre-pregnancy BMI, gravidity, parity, gestational age at delivery, weight gain during pregnancy, cigarette smoke and alcohol consumption pre-pregnancy. A value of P < 0.05 was regarded as statistically significant.

## Results

Of the 722 women with PCOS included in the study, 83.5% were primiparas, and 16.5% were multiparas. The average maternal age (± SD) was 31.7 ± 6.1 years, gestational age was 38.7 ± 1.7 weeks, and the average pre-pregnancy BMI was 23.6 ± 1.7 kg/m^2^. Among the 722 subjects, 47 (6.5%), 460 (63.7%), 152 (21.1%), and 63 (8.7%) were underweight, normal weight, overweight, and obese, respectively, prior to pregnancy. Approximately 169 (23%), 323 (45%), and 230 (32%) women experienced inadequate, adequate, and excessive weight gain during pregnancy, respectively, according to the 2009 IOM guidelines. Analysis of the pregnancy outcomes revealed that 22.6%, 12.3%, 5.4%, and 10.9% of the study subjects developed GDM, GH, PE, and PPH, respectively. Regarding the mode of delivery, 56.8%, 8.3%, and 34.9% of the women required vaginal, assisted vaginal, and cesarean section delivery, respectively. Furthermore, 8.7%, 1.8%, and 18.8% of the newborns were macrosomia, SGA, and LGA, respectively.

Table 2 shows the relationship between pre-pregnancy BMI and the pregnancy outcomes. Compared with the women with normal weight before pregnancy, underweight women were positively correlated with the risk of SGA birth (OR 12.35, 95% CI 3.56–42.82) and vaginal delivery (OR 2.21, 95% CI 1.09–4.50), but were negatively correlated with the risk of LGA (OR 0.21, 95% CI 0.05–0.88). Overweight and obese women had increased risk of developing gestational hypertension (OR 4.86, 95% CI 2.82–8.39; and OR 6.05, 95% CI 2.97–12.33, respectively), undergoing cesarean section delivery (OR 1.71, 95% CI 1.15–2.55; and OR 2.10, 95% CI 1.18–3.74, respectively), and having an infant with LGA (OR 2.57, 95% CI 1.64–4.04; and OR 2.22, 95% CI 1.12–4.39, respectively). Overweight women were more likely to develop PE (OR 4.08, 95% CI 1.95–8.51) and result in macrosomia at birth (OR 2.15, 95% CI 1.14–4.05) when compared with normal weight PCOS women.

**Table 2:** The relationship between BMI categories and maternal/fetal outcomes

Outcome	Underweight	Overweight	Obesity
	Adjusted OR 95% CI	Adjusted OR 95% CI	Adjusted OR 95% CI
GH	0.24 [0.03–1.80]	4.86 [2.82–8.39]**	6.05 [2.97–12.33]**
PE	0.55 [0.07–4.29]	4.08 [1.95–8.51]**	1.46 [0.40–5.37]
GDM	0.56 [0.21–1.51]	1.33 [0.85–2.09]	1.23 [0.65–2.32]
PPH	0.18 [0.02–1.35]	1.60 [0.91–2.81]	1.88 [0.83–4.24]
Cesarean section	0.55 [0.25–1.20]	1.71 [1.15–2.55]*	2.10 [1.18–3.74]*
Assisted vaginal delivery	0.36 [0.08–1.56]	0.50 [0.23–1.12]	0.41 [0.12–1.42]
Vaginal delivery	2.21 [1.09–4.50]*	0.75 [0.51–1.09]	0.65 [0.37–1.14]
Macrosomia	0.46 [0.10–2.07]	2.15 [1.14–4.05]*	1.54 [0.52–4.55]
SGA	12.35 [3.56–42.82]**	0.41 [0.05–3.62]	NS
LGA	0.21 [0.05–0.88]*	2.57 [1.64–4.04]**	2.22 [1.12–4.39]*

Data was analyzed using multivariable logistic regression analysis. Models were adjusted for maternal age, height, gravidity, parity, gestational age at delivery, weight gain during pregnancy, cigarette smoke pre-pregnancy, and alcohol consumption pre-pregnancy

Reference group: normal weight for pre-pregnancy

*OR* odds ratio, *CI* confidence interval, *GH* gestational hypertension, *PE* preeclampsia, *GDM* gestational diabetes mellitus, *PPH* postpartum hemorrhage, *SGA* small for gestational age, *LGA* large for gestational age, *NS* the number in this category was too small to analyze

^*^P < 0.05

**P < 0.001

As Table 3 shows, significantly lower (P < 0.05) incidences of GH and PPH were observed in the inadequate GWG groups, whereas significantly higher (P < 0.001) LGA and macrosomia incidences were reported in the excessive GWG group relative to the other 2 groups. The results from multivariate logistic regression demonstrated that compared with the women in the adequate GWG group, individuals in the inadequate GWG group had a lower risk of developing GH (OR 0.28, 95% CI 0.12–0.66) and PPH (OR 0.38, 95% CI 0.17–0.84), while those in the excessive GWG group were more likely to deliver macrosomia (OR 1.93, 95% CI 1.05–3.54) and give birth to LGA infants (OR 1.94, 95% CI 1.27–2.96). Further subgroup analyses based on stratification of body mass indices revealed that when compared with the adequate GWG group women with the same BMI, inadequate GWG decreased the risks of GH (OR 0.24, 95% CI 0.08–0.71) and PPH (OR 0.38, 95% CI 0.16–0.95) in women with BMI < 25 kg/m^2^ before pregnancy (Table 4). Moreover, excessive GWG was related to the higher possibility of LGA birth (OR 2.41, 95% CI 1.40–4.18) in women with BMI < 25 kg/m^2^, as well as the likelihood of undergoing a cesarean section (OR 2.06, 95% CI 1.01–4.20) in women with BMI ≥ 25 kg/m^2^ (Table 5).

**Table3:** The relationship between GWG categories and maternal/fetal outcomes

Outcome	Pregnancy outcomes by gestational weight gain category				Multivariable logistic regression analyses	
	Inadequate (N = 169)	Adequate (N = 323)	Excessive (N = 230)	P-value	Inadequate weight gain	Excessive weight gain
GH (N)	7 (4.1%)	42 (13%)	40 (17.4%)	< 0.001	0.28 [0.12–0.66]	1.15 [0.69–1.91]
PE (N)	5 (3%)	17 (5.3%)	17 (7.4%)	0.15	0.56 [0.20–1.56]	1.36 [0.66–2.78]
GDM(N)	64 (37.9%)	69 (21.4%)	30 (13%)	< 0.001	2.30 [1.49–3.54]	0.49 [0.30–0.80]
PPH (N)	8 (4.7%)	38 (11.8%)	33 (14.3%)	0.008	0.38 [0.17–0.84]	1.21 [0.73–2.04]
Cesarean section (N)	56 (33.1%)	108 (33.4%)	88 (38.3%)	0.43	0.88 [0.58–1.34]	1.28 [0.88–1.87]
Assisted vaginal delivery (N)	14 (8.3%)	30 (9.3%)	16 (7.0%)	0.62	0.97 [0.49–1.92]	0.64 [0.34–1.24]
Vaginal delivery (N)	99 (58.6%)	185 (57.3%)	126 (54.8%)	0.73	1.13 [0.76–1.67]	0.91 [0.64–1.29]
Macrosomia (N)	7 (4.1%)	22 (6.8%)	34 (14.8%)	< 0.001	0.67 [0.27–1.66]	1.93 [1.05–3.54]
SGA (N)	4 (2.4%)	5 (1.5%)	4 (1.7%)	0.72	1.23 [0.31–4.87]	1.25 [0.32–4.95]
LGA (N)	18 (10.7%)	53 (16.4%)	72 (31.3%)	< 0.001	0.65 [0.36–1.17]	1.94 [1.27–2.96]

Multivariable logistic regression analysis was adjusted for maternal age, height, gravidity, parity, gestational age at delivery, pre-pregnancy BMI, cigarette smoke pre-pregnancy, and alcohol consumption pre-pregnancy

Reference group: adequate GWG group

*N* number of cases, *GH* gestational hypertension, *PE* preeclampsia, *GDM* gestational diabetes mellitus, *PPH* postpartum hemorrhage, *SGA* small for gestational age, *LGA* large for gestational age

**Table 4:** Pregnancy outcomes among women whose weight gain was below recommended levels by guidelines of the Institute of Medicine

Outcome	BMI < 25 kg/m^2^		BMI ≥ 25 kg/m^2^	
	Adjusted OR 95% CI	P-value	Adjusted OR 95% CI	P-value
GH	0.24 [0.08–0.71]	0.01	0.27 [0.05–1.33]	0.11
PE	0.62 [0.19–2.01]	0.42	NS	
GDM	2.55 [1.54–4.23]	< 0.001	2.16 [0.87–5.38]	0.10
PPH	0.38 [0.16–0.95]	0.04	0.22 [0.02–1.95]	0.17
Cesarean section	0.83 [0.52–1.33]	0.43	0.94 [0.38–2.34]	0.89
Assisted vaginal delivery	0.88 [0.42–1.84]	0.73	1.18 [0.14–10.02]	0.88
Vaginal delivery	1.23 [0.79–1.92]	0.35	1.06 [0.43–2.63]	0.90
Macrosomia	0.78 [0.28–2.14]	0.63	0.26 [0.02–3.37]	0.30
SGA	1.43 [0.36–5.67]	0.61	NS	
LGA	0.54 [0.25–1.16]	0.11	1.05[0.34–3.23]	0.93

Data was analyzed using multivariable logistic regression analysis. Models were adjusted for maternal age, height, gravidity, parity, gestational age at delivery, cigarette smoke pre-pregnancy, and alcohol consumption pre-pregnancy

Reference group: adequate GWG in the same BMI category

*OR* odds ratio, *CI* confidence interval, *GH* gestational hypertension, *PE* preeclampsia, *GDM* gestational diabetes mellitus, *PPH* postpartum hemorrhage, *SGA* small for gestational age, *LGA* large for gestational age, *NS* the number in this category was too small to analyze

**Table 5:** Pregnancy outcomes among women whose weight gain was above recommended levels by guidelines of the Institute of Medicine

Outcome	BMI < 25 kg/m^2^		BMI ≥ 25 kg/m^2^	
	Adjusted OR 95% CI	P-value	Adjusted OR 95% CI	P-value
GH	0.43 [0.17–1.11]	0.08	1.85 [0.86–4.00]	0.12
PE	0.48 [0.13–1.79]	0.27	2.08 [0.69–6.32]	0.20
GDM	0.31 [0.14–0.72]	0.006	0.56 [0.27–1.17]	0.12
PPH	1.15 [0.57–2.30]	0.70	1.50 [0.70–3.21]	0.29
Cesarean section	0.94 [0.57–1.54]	0.80	2.06 [1.01–4.20]	0.048
Assisted vaginal delivery	0.88 [0.42–1.83]	0.72	0.43 [0.07–2.74]	0.37
Vaginal delivery	1.10 [0.70–1.73]	0.69	0.56 [0.28–1.12]	0.10
Macrosomia	1.90 [0.87–4.17]	0.11	2.17 [0.69–6.79]	0.19
SGA	1.29 [0.28–5.82]	0.74	NS	
LGA	2.41 [1.40–4.18]	0.002	1.53[0.73–3.23]	0.26

Data was analyzed using multivariable logistic regression analysis. Models were adjusted for maternal age, height, gravidity, parity, gestational age at delivery, cigarette smoke pre-pregnancy and alcohol consumption pre-pregnancy

Reference group: adequate GWG in the same BMI category

*OR* odds ratio, *CI* confidence interval, *GH* gestational hypertension, *PE* preeclampsia, *GDM* gestational diabetes mellitus, *PPH* postpartum hemorrhage, *SGA* small for gestational age, *LGA* large for gestational age, *NS* the number in this category was too small to analyze

## Discussion

Our results showed a positive association between women who were underweight, prior to pregnancy, with the incidence of SGA infants, and a negative relationship between this group of women with the incidence of LGA infants. We also found that pre-pregnancy overweight but not obesity was more susceptible to developing PE. Furthermore, GWG below the recommended level significantly reduced the risk for GH, and PPH in women with pre-pregnancy BMI < 25 kg/m^2^, whereas that above the recommended threshold increased the chances of cesarean section in those with a pre-pregnancy BMI ≥ 25 kg/m^2^. For women with BMI ≥ 25 kg/m^2^, GWG below the recommendation did not reduce the likelihood of LGA, and GWG above the recommendation did not reduce the probability of SGA in women with BMI < 25 kg/m^2^.

Our findings further indicated that pre-pregnant underweight PCOS women were at a significantly higher risk of SGA but at a lower risk for LGA, relative to normal weight PCOS counterparts. Particularly, underweight PCOS women had a 12-fold risk of having SGA compared with normal weight counterparts, which was much higher than general underweight women in China and Lebanon (OR 1.67, 95% CI 1.49–1.87 and OR 1.50, 95% CI 1.37–1.63, respectively) [15, 16]. PCOS in pregnancy can involve elevated androgen concentration level, which might affect fetal outcomes [17]. Therefore, the superposition effect of PCOS and underweight state may contribute to this result. Furthermore, GWG above the recommendation exhibited a significantly positive correlation with LGA birth in women with BMI < 25 kg/m^2^, compared with GWG within the IOM guidelines in the same BMI category. Therefore, these results indicate that women with a lower BMI could adhere to the IOM guidelines to obtain optimal fetal growth, since higher weight gain does not guarantee better pregnancy outcomes in this group of women.

In the present study, both overweight and obesity conditions increased the risk of GH, but showed different effects on PE. Specifically, PE was associated with overweight but not obese women, which was contrary to previous reports. For example, a study conducted in Belgium found no significant differences in the prevalence of GH and preeclampsia between overweight and normal weight PCOS women [18]. The differences in findings could be possibly due to races and potential confounders. On the other hand, a study on general Chinese pregnant women revealed that underweight, overweight, and obese conditions increase the risk of GH [7], but they did not investigate whether various BMI has an impact on developing PE. Our results revealed that both overweight and obese PCOS women were more likely to give birth to LGA infants, whereas maternal overweight conditions increased the risk of macrosomia compared with normal weight women. However, there was no significant relationship between obesity and macrosomia, consistent with a previous retrospective study, which indicated that high BMI had no significant impact on the risk of delivering LGA newborn or macrosomia, in PCOS women who underwent frozen embryo transfer [19]. A study involving general pregnant Chinese women found that overweight and obese women were more prone to have LGA and macrosomia compared with normal weight counterparts [20]. A retrospective study involving 7122 general pregnant women from Bratislava, Slovakia, revealed that women with overweight and obesity had a 1.7- to 1.8-fold risk of macrosomia compared with normal weight women [21], but the study did not examine the correlation between BMI and LGA birth. It might be partly that different ways of conception may have specific effects on pregnancy outcomes.

Based on the 2009 IOM guidelines, 45% of the PCOS women in the present study achieved adequate weight gain during pregnancy, which was higher than the previously reported frequencies, including 29.6 and 30% in general and PCOS women, respectively (22, 23). This may be attributed to strict management regimes given to PCOS women at our hospital. Specifically, once PCOS women became pregnant, they were admitted to a specialized outpatient section where they received individualized medical nutritional therapy (MNT), as well as exercise guidance to help control weight gain during pregnancy.

We evaluated the effect of different GWG on PCOS women with BMI ≥ 25 kg/m^2^, However, we did not establish any relationship between various GWG and fetal growth in this BMI group. This is possibly related to the sample size in our study. On the other hand, GWG may play a relatively weak role in fetal growth. Therefore, this implies that more effort should be shifted to pre-conceptional weight management in PCOS women to achieve a normal weight. Previous studies have shown that overweight/obesity and PCOS are risk factors for GDM [24, 25]. In addition, early evidence suggests that clinical features of PCOS, such as polycystic ovaries, insulin resistance, and hyperandrogenism, might be potential factors of the GDM [26]. In the present study, we found a high incidence of GDM in overweight (30.2%) and obese (36.5%) PCOS women, relative to normal weight (19.8%) counterparts, however, with no statistical significance following multivariate regression analysis. This could be attributed to the intervention for PCOS women before or during pregnancy. Moreover, different diagnostic criteria and the presence of heterogeneity between study populations could explain this.

From our results, it is evident that inadequate GWG is a protective factor for GH and PPH in women with pre-pregnancy BMI < 25 kg/m^2^, relative to those whose weight gain is within the optimal range in the same BMI category. However, we found no impact on overweight and obese pregnant women, indicating that GWG has different effects on GH and PPH across different BMI groups. In the future, large sample sized and multi-center studies should be conducted to validate these findings. Our results also indicated a positive relationship between excessive GWG and the incidence of cesarean section in women with BMI ≥ 25 kg/m^2^, relative to those with weight gain within the IOM guidelines in the same BMI category. This was consistent with a previous study that associated high GWG with cesarean delivery in women with obesity class I (BMI 30.0–34.9 kg/m^2^) compared with those who met gestational weight gain goals [27]. However, for women with BMI ≥ 25 kg/m^2^, inadequate GWG did not reduce the risk of LGA birth, which was consistent with our previous results showing that excessive GWG has no protective role in delivering an SGA newborn in women with BMI < 25 kg/m^2^. Our finding was inconsistent with the previous studies involving general pregnant women [7, 28]. This could be attributed to the small number of SGA cases in our study. Besides, whether the factors entailing complicated endocrine and metabolism are involved in PCOS should be further investigated. Moreover, we found an inverse relationship between GWG and GDM, when PCOS women were diagnosed with GDM at 24–28 weeks’ gestation, consistent with the findings of previous studies [7, 29]. To control weight gain and blood sugar levels, these women may undergo MNT, acquire exercise guidance, and insulin therapy when necessary, hence, the real association between GWG and GDM may have been masked. Therefore, further research is required to confirm the relationship between weight gain and the development of GDM among pregnant women with PCOS at different pregnancy periods.

Our study had several limitations. First, the pre-pregnancy body weights of the subjects were self-reported, at the first visit (week 6–8 or so). It is possible that recall bias may have occurred, thereby affecting the evaluation of BMI and GWG. Secondly, our study population mainly comprised subjects from the Beijing area, implying that our findings cannot be generalized to individuals from other Chinese regions or countries, because of the potential differences in education, socio-economic levels, as well as varying environmental factors.

## Conclusions

Our findings indicated that the correlations between pre-pregnancy BMI, GWG, and pregnancy outcomes among PCOS women, observed herein were similar to what has been previously reported in general women. However, some unique trends existed in PCOS women. Specifically, being underweight significantly increased the risk of SGA birth, overweight but not obesity was correlated with the risk of PE. Conversely, overweight or obese conditions were not associated with GDM, whereas inadequate GWG was a protective factor for GH, and PPH only in women with pregestational BMI < 25 kg/m^2^. Inadequate GWG did not reduce the possibility of LGA birth in women with BMI ≥ 25 kg/m^2^ and excessive GWG did not decrease the risk for SGA in women with BMI < 25 kg/m^2^. In general, pre-pregnancy BMI and GWG were shown to have different impacts on pregnancy outcomes between general and PCOS pregnant women. Taken together, these findings suggest that the management/intervention for PCOS women should focus on pre-conceptional weight management. Future studies should elucidate the ideal pre-pregnancy weight and ascertain the methods for appropriate weight gain during pregnancy.

## Data Availability

The data is available upon reasonable request to the corresponding author.

## Abbreviations

PCOS: Polycystic ovary syndrome
BMI: Body mass index
GWG: Gestational weight gain
SGA: Small for gestational age
LGA: Large for gestational age
PPH: Postpartum hemorrhage
GDM: Gestational diabetes mellitus
PE: Preeclampsia
GH: Gestational hypertension
WHO: World Health Organization
ESHRE: European Society of Human Reproduction and Embryology
IOM: Institute of Medicine
MNT: Medical nutritional therapy
OR: Odds ratio
CI: Confidence interval
SD: Standard deviations
